# Supplementary material for: CUDASW++4.0: ultra-fast GPU-based Smith–Waterman protein sequence database search
Source: BMC Bioinformatics. 2024 Nov 2;25:342. doi: 10.1186/s12859-024-05965-6 (PMC11531700; doi:10.1186/s12859-024-05965-6)
Supplement: Supplementary file 1 [file 12859_2024_5965_MOESM1_ESM.pdf]

CUDASW++4.0: Supplementary File

Bertil Schmidt, Felix Kallenborn, Alejandro Chacon, Christian Hun

January 2024

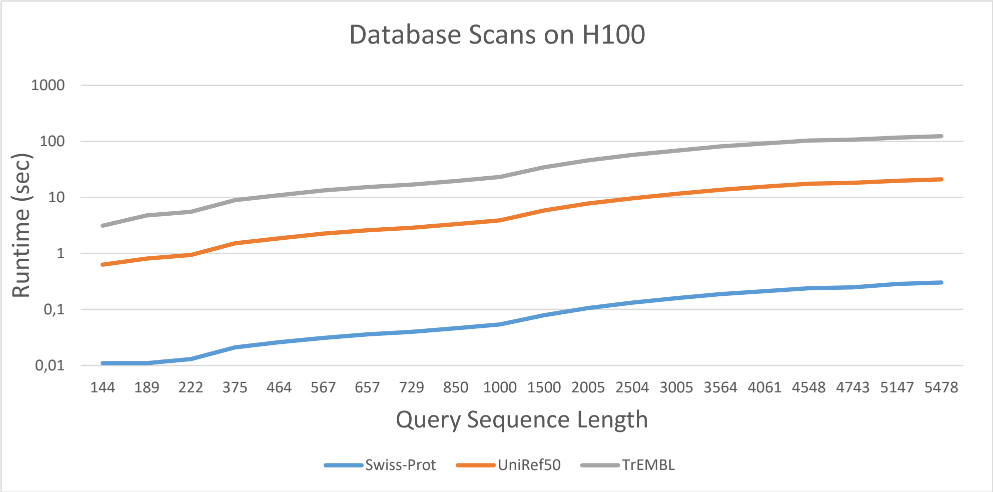

Figure 1: CUDASW++4.0 runtimes (in seconds) for scanning Swiss-Prot, UniRef50, and TrEMBL with queries of different length on H100.

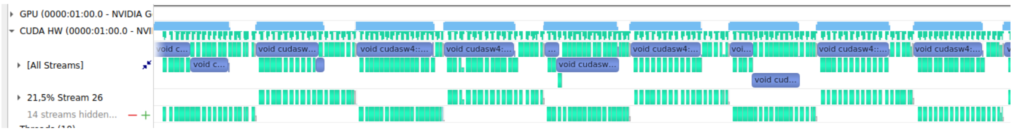

Figure 2: Screenshot of the Nsight systems profiler for execution of CUDASW++4.0 using a query of length 222 to scan a batched UniRef50 database.

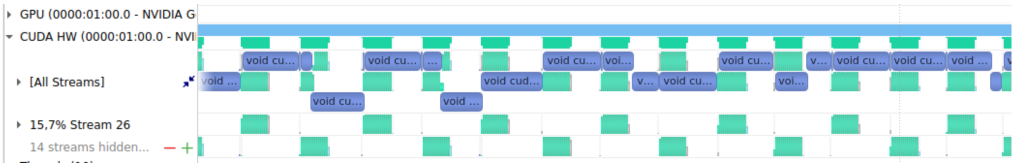

Figure 3: Screenshot of the Nsight systems profiler for execution of CUDASW++4.0 using a query of length 375 to scan a batched UniRef50 database.
